# Is Bariatric Surgery a Prophylaxis for Pelvic Floor Disorders?

**DOI:** 10.1007/s11695-017-3067-x

**Published:** 2017-12-18

**Authors:** Andrzej Pomian, Wojciech Majkusiak, Wojciech Lisik, Paweł Tomasik, Edyta Horosz, Aneta Zwierzchowska, Jacek Kociszewski, Ewa Barcz

**Affiliations:** 1 1st Department of Obstetrics and Gynecology, Medical University of Warsaw, Pl. Starynkiewicza 1/3, 02-015 Warsaw, Poland; 2 Department of General and Transplantation Surgery, Medical University of Warsaw, Warsaw, Poland; 3 Evangelisches Krankenhaus Hagen-Haspe, Hagen, Germany

**Keywords:** Bariatric surgery, Pelvic organ prolapse, Pelvic floor disorders, Urethral mobility, Female incontinence, Obesity

## Abstract

**Introduction:**

Obesity is one of the well-documented risk factors of pelvic floor disorders (PFDs). The PFDs include urinary and fecal incontinence (UI, FI) and pelvic organ prolapse (POP). Surgery-induced weight loss improves different kinds of incontinence as well as POP symptoms. However, there is a lack of evidence how bariatric surgery influences pelvic floor anatomy and function in women without previous PFDs and whether it may be concerned as PFD prophylaxis tool.

**Materials and Methods:**

The present analysis is a prospective, non-randomized case-control study from January 2014 to September 2017. Participants underwent pelvic floor ultrasound examination with bladder neck position estimation at rest, during levator ani tension, and at Valsalva maneuver before surgery and 12–18 months after. Pelvic organ prolapse quantification (POPQ) > 2 stage and PFD complaints were the exclusion criteria.

**Results:**

Fifty-nine patients underwent bariatric surgery (57 sleeve gastrectomy and 2 gastric bypass). Mean BMI decreased from 43.8 ± 5.9 to 29 ± 4.6 kg/m^2^ after surgery (*p* < 0.001). Statistically significant higher position of the bladder neck at rest, during tension, and at Valsalva maneuver (*p* < 0.05) was shown after surgery. We did not demonstrate differences in bladder neck mobility and bladder neck elevation at tension after weight loss.

**Conclusions:**

Bariatric surgery is associated with a betterment of bladder neck position at rest, tension, and Valsalva maneuver in women without PFDs. We postulate that bariatric surgery may be a tool for PFD prevention. It does not improve levator ani function and does not limit bladder neck mobility, which implicates that it has no influence on preexisting pelvic dysfunction.

## Introduction

Obesity is defined as a body mass index (BMI) greater than 30 kg/m^2^. It is a worldwide public health problem as it has a negative impact on the individual’s well-being and is a risk for many chronic diseases (metabolic syndrome, musculoskeletal disorders, and certain types of cancer). The prevalence of obesity has doubled within the last three decades and currently almost 15% of the world population is obese [1].

Pelvic floor disorders (PFDs) encompass a broad spectrum of health problems, including different types of urinary incontinence (UI), pelvic organ prolapse (POP), fecal incontinence (FI), and defecatory and sexual dysfunctions. PFDs influence medical, emotional, social, and economic issues of women all over the world. It is estimated that different conditions connected with pelvic floor disorders concern approximately 30% of adult women population worldwide with increased incidence in elderly and obese population [2].

There are many risk factors for developing pelvic floor disorders including vaginal and instrumental deliveries, age, race, family history, and last but not least, overweight and obesity [3]. Women with obesity are at much higher risk as compared to normal-weight individuals for developing different types of incontinence and POP. It is estimated that over 50% of women with a BMI greater than 35 kg/m^2^ report a PFD, compared with approximately 30% of women with a normal body mass index [4].

There are strong evidences that reducing weight improves urinary incontinence. It was shown that after bariatric surgery, there were significant improvements in voiding status assessed by voiding questionnaires [5], as well as in objective tests such as pad test [6]. Additionally, it was confirmed that weight loss after surgery improves various lower urinary tract symptoms such as stress urinary incontinence, urge incontinence, and dysuria, as well as quality of life in the above aspect [7]. It was also shown on the basis of patients’ questionnaires that bariatric surgery improves different symptoms related to pelvic floor disorders connected with POP (prolapse, lower urinary tract, colorectal symptoms, and sexual dysfunctions) [8, 9].

Most current studies on the influence of bariatric surgery on PFDs focus on subjective improvement of symptoms, basing on different kinds of questionnaires and rarely rate objective signs of pelvic floor anatomy and function. Moreover, authors usually concentrate on already existing symptoms of PFDs. Till now, there is no data if and how weight loss may influence pelvic floor anatomy and/or function in women without PFDs and whether it may serve as a prophylaxis for future possible failure.

The urethro-vesical junction (bladder neck) is a point that corresponds to point Aa in POPQ (pelvic organ prolapse quantification scale) on the anterior vaginal wall 3 cm from the vaginal vestibule. Lowering of its position is connected with anterior vaginal wall descent as well as with higher risk of urinary incontinence. Bladder neck hypermobility (the difference between bladder neck positions at rest vs during Valsalva maneuver) is connected with higher incidence of stress UI [10] whereas the levator ani injury may be the cause of bladder neck descent and future pelvic floor dysfunctions and prolapse [11]. Elevation of the bladder neck during tension is realized by contraction of the most important muscle of the pelvic floor—levator ani. The measurement of the above parameter may serve as an indirect indicator of its function. The aim of the present study was to evaluate whether BMI reduction after bariatric surgery influences bladder neck position as well as its mobility and levator ani function in obese women without previous history of pelvic floor dysfunction.

## Material and Methods

The present analysis is a prospective non-randomized single-center case-control study, approved by local ethic committee before initiation.

Fifty-nine adult obese women (BMI > 35) who were scheduled for bariatric surgery were included in the trial. The inclusion criteria were obesity, no PFD symptoms, and POPQ examination < 2 within all compartments. All patients underwent pelvic floor ultrasound examination with evaluation of three parameters: bladder neck position at rest, during levator ani tension, and during maximum Valsalva maneuver. The control examination took place minimum 1 year after bariatric surgery.

Bladder neck position was measured in a standardized manner, with the patient on the gynecological chair in a semi sitting position with the bladder filled to 200–400 ml (the association between the bladder filling and bladder neck position in the volume range of 200–400 ml was not statistically significant). Three diameters of the bladder was measured in order to estimate the bladder volume at the beginning of the examination. The probe (a 3.6–8.3-MHz vaginal transducer with a beam angle of 160°) was placed in the vaginal introitus at the level of the external urethral orifice. With the probe in this position, the bladder neck (BN), urethra (U), and pubic symphysis (PS) with the interpubic disc were visualized in the median sagittal plane, according to Interdisciplinary S2k Guideline: Sonography in Urogynecology [12].

**Fig. 1:**
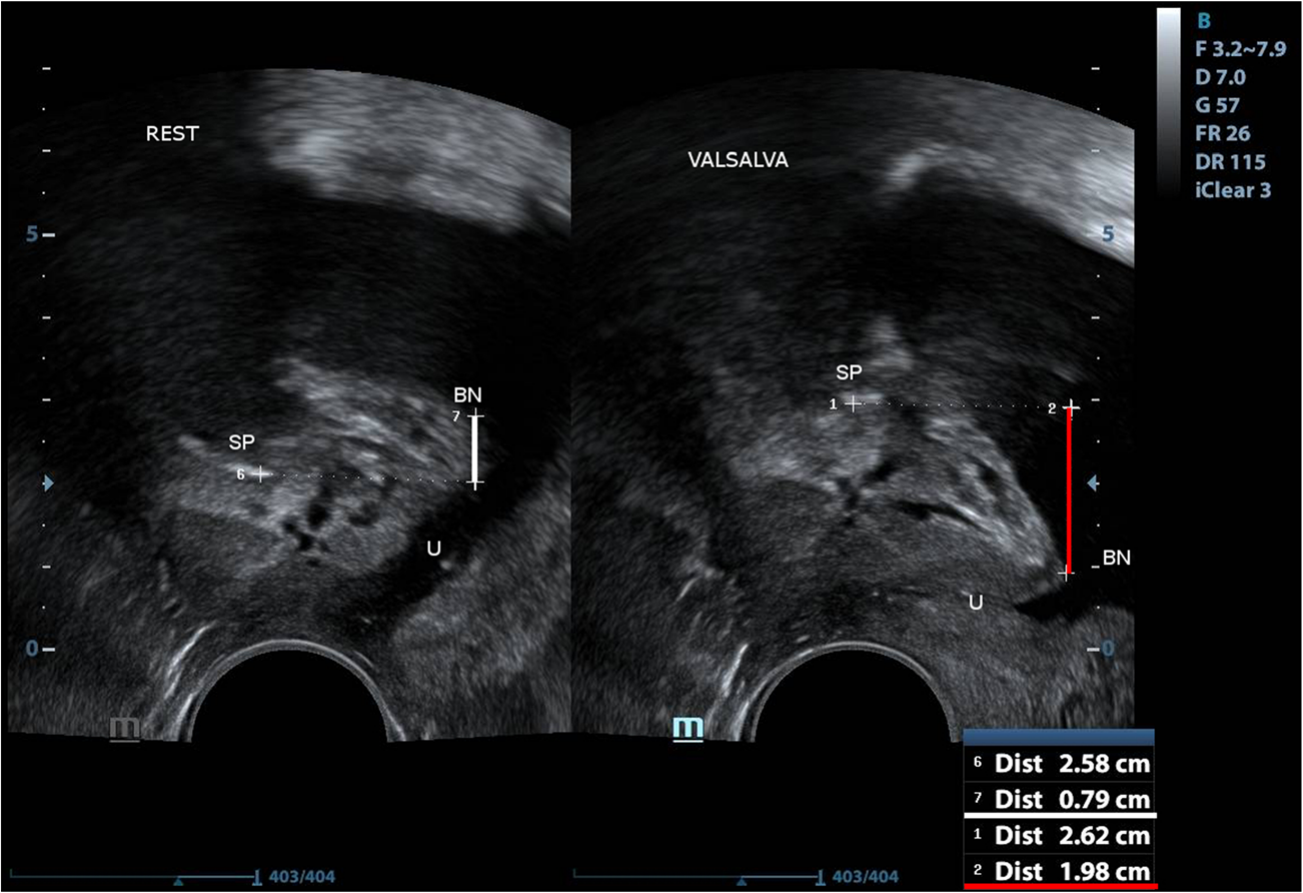
Measurement of bladder neck position at rest: the shortest distance between the point of urethral-bladder junction and the horizontal line running through the lower edge of symphysis pubis (marked with white line) and bladder neck position measurement in maximal descent point during Valsalva maneuver (marked with the red line). SP symphysis pubis, U urethra, BN bladder neck

**Fig. 2:**
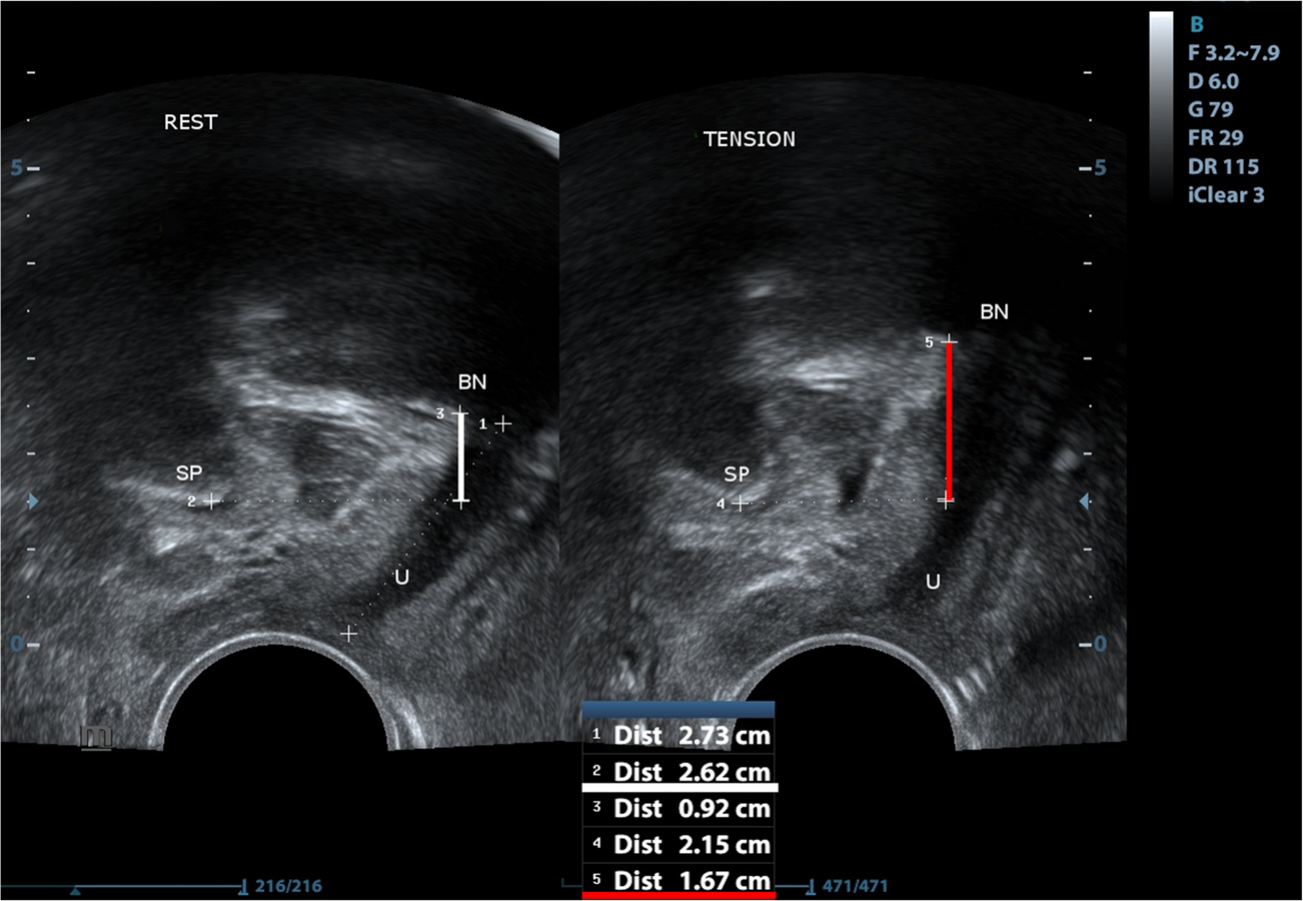
Measurement of bladder neck position at rest: the shortest distance between the point of urethral-bladder junction and the horizontal line running through the lower edge of symphysis pubis (marked with the white line) and bladder neck position measurement in maximal elevation point at contracting levator ani muscle (marked with the red line). SP symphysis pubis, U urethra, BN bladder neck

Bladder neck position at rest was measured as the shortest distance between the point of urethral-bladder junction and the horizontal line running through the lower edge of symphysis pubis and was shown in millimeters. Accordingly, bladder neck position was measured in maximal descent point during Valsalva maneuver (Fig. 1) and in maximal elevation point at contracting levator ani muscle (Fig. 2).

Bladder neck mobility was defined as the difference between its position at rest and during Valsalva maneuver and was shown in millimeters. Bladder neck elevation during contraction was shown as the difference between bladder neck position at rest and during levator ani muscle tension.

Descriptive statistical analysis and statistical tests were performed using the R version 3.4.0 (by the R Foundation for Statistical Computing). Normality was tested using Lilliefors and Shapiro-Wilk *W* tests. We associated the degree and type of non-adherence using the Wilcoxon signed-rank test and multivariate variance analysis (MANOVA) and multiple regression for multivariable analysis. We established a significance level of *p* < 0.05.

## Results

Fifty-nine women without PFDs were enrolled in the study. Demographic features of the study group are shown in Table 1.

**Table 1:** Demographic features of examined group

	Before surgery	After surgery
Age	42.2 ± 11.8	43.5 ± 11.5
BMI	43.7 ± 5.8	29 ± 4.6
%EWL	81.3 ± 22.9%	
%total body weight loss	33.9 ± 8.7%	
Parity	1.5 ± 1.3	
Nulliparas	32.2%	
Menopause	32.2%	
Surgery type	57 patients—SG 2 patients—RYGB	

*BMI* body mass index, *%EWL* %excess weight loss, *SG* sleeve gastrectomy, *RYGB* Roux-en-Y gastric bypass

In all the examined cases, significant weight loss was observed after bariatric surgery (43.7 ± 5.8 vs 29 ± 4.6 kg/m^2^).

**Fig. 3:**
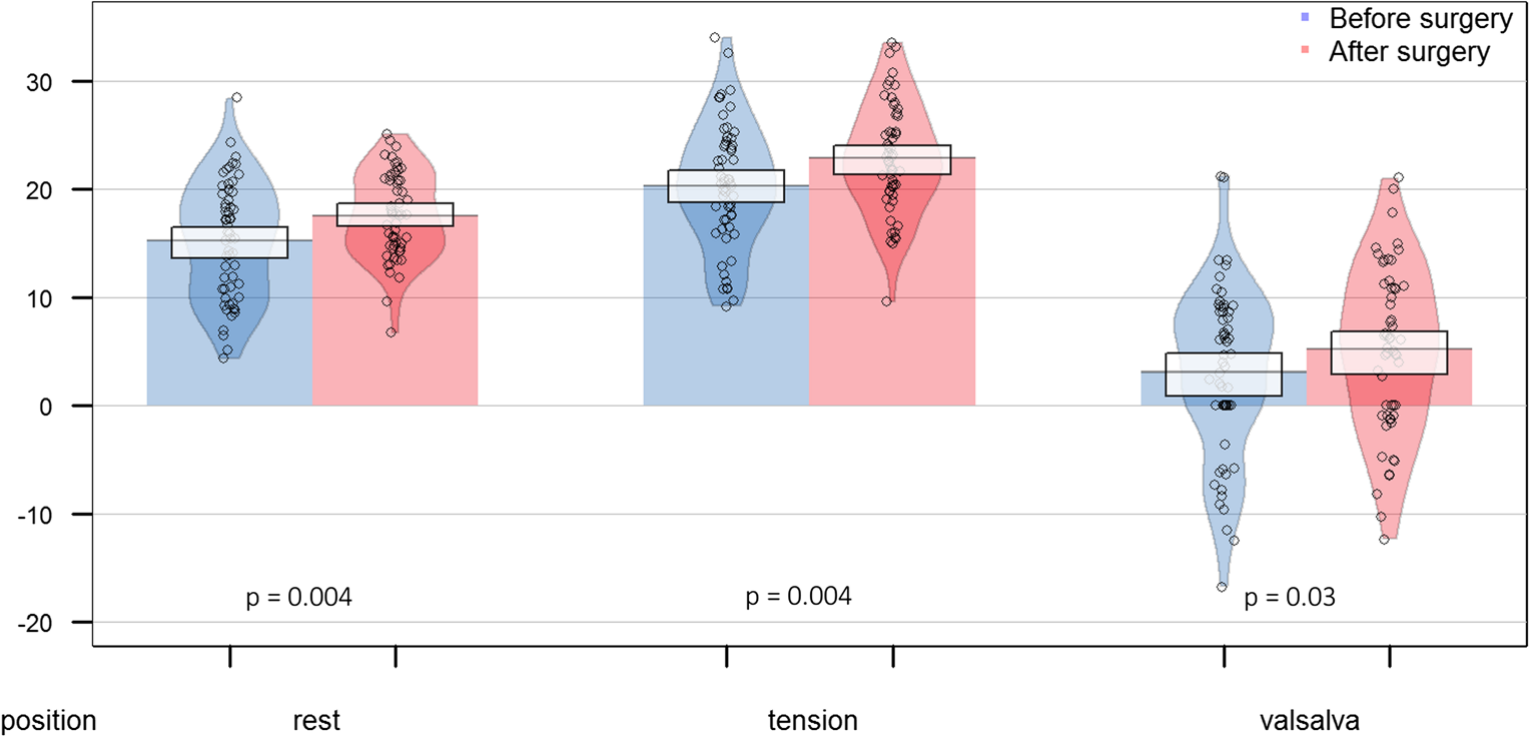
Bladder neck position at rest, tension, and during Valsalva maneuver before and after bariatric surgery with marked values density and 0.95 confidence interval (*n* = 59)

We showed statistically significant elevation of the bladder neck position at rest in patients who underwent bariatric surgery (*p* = 0.004) 15.2 ± 5.4 vs 17.6 ± 4.0 mm. Significantly higher position of the bladder neck at tension (*p* = 0.004) 20.3 ± 5.7 vs 22.9 ± 5.1 mm as well as during Valsalva maneuver (*p* = 0.03) 3.0 ± 7.9 vs 5.1 ± 7.7 mm was observed after weight loss (Fig. 3).

Age, parity, mode of delivery, and hormonal status did not influence the observed changes in bladder neck position at rest, tension, and Valsalva maneuver in multivariable analysis.

**Fig. 4:**
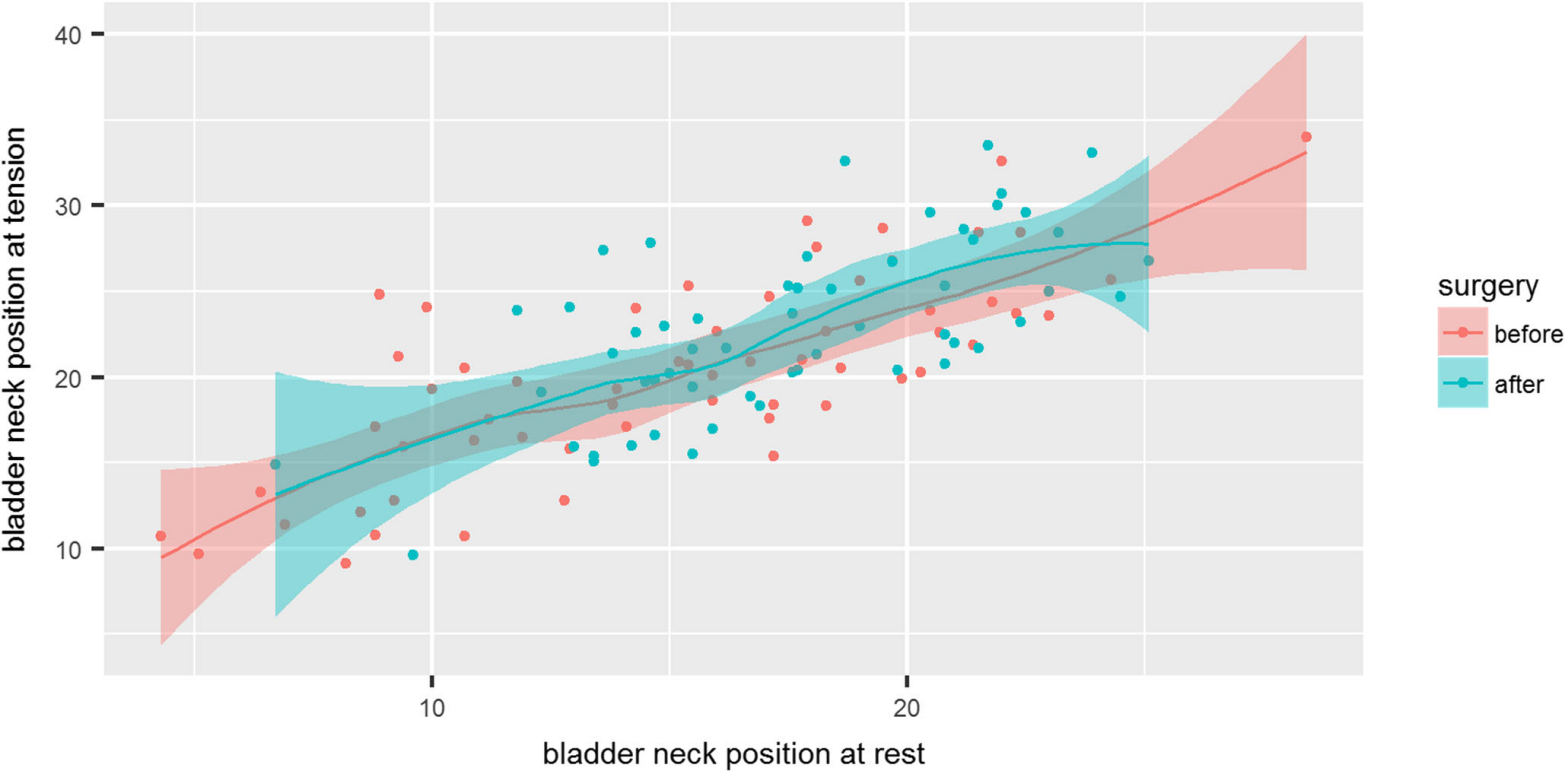
Scatterplot of bladder neck position at rest vs at tension before and after surgery with marked 0.95 confidence interval. The degree of inclination of the trend line corresponds to the levator ani function before (red) and after surgery (green) and does not differ significantly

The calculation of absolute value of change of the bladder neck position at rest vs at levator ani tension showed no differences in muscle function following weight lost after bariatric surgery 5.1 ± 3.8 vs 5.4 ± 3.8 (n/s, *p* = 0.94) (Fig. 4).

**Fig. 5:**
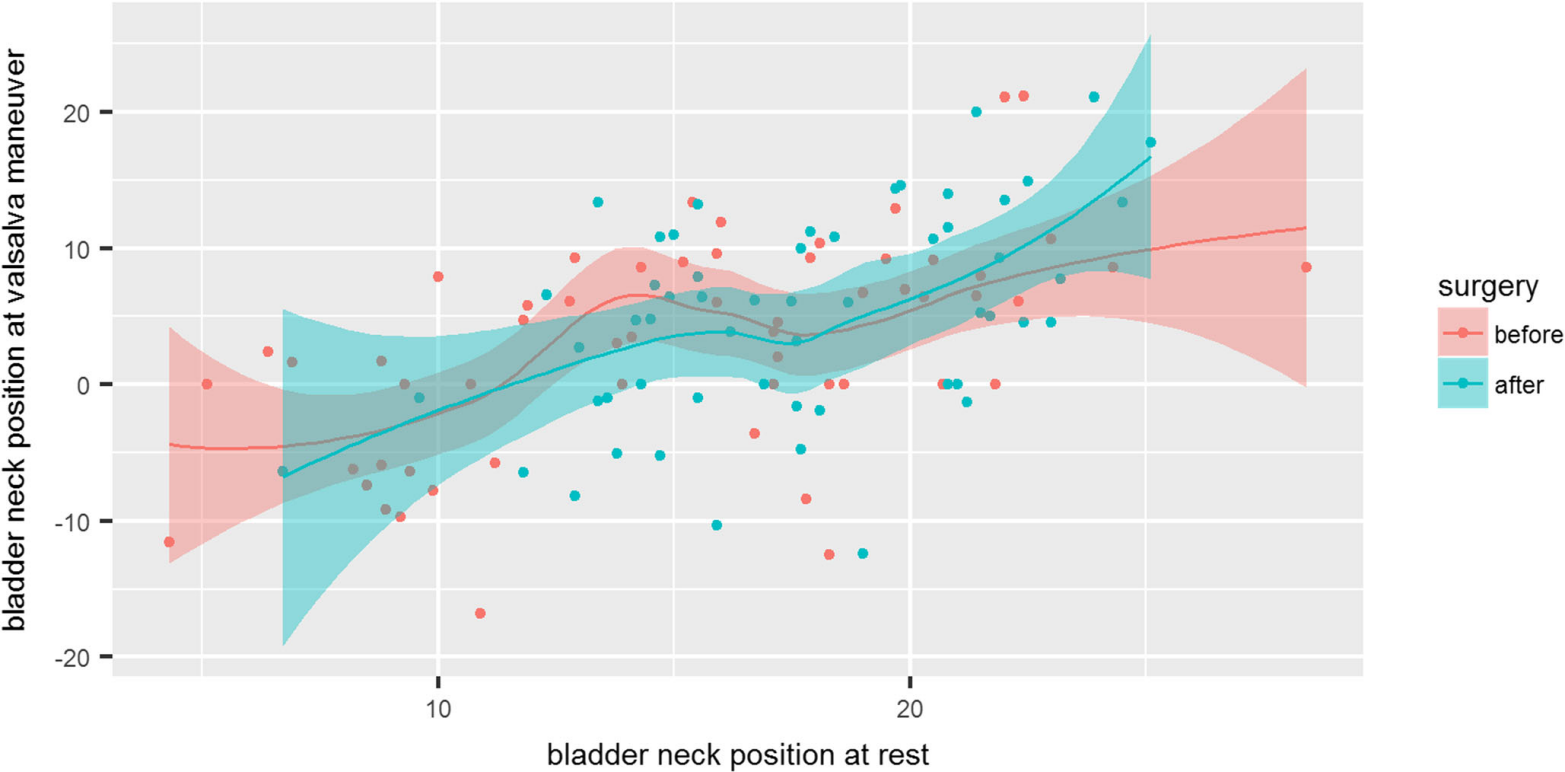
Scatterplot of bladder neck position at rest vs at tension before and after surgery with marked 0.95 confidence interval. The degree of inclination of the trend line corresponds to the urethral mobility before (red) and after surgery (green) and does not differ significantly

Similarly, we did not show changes in bladder neck mobility after bariatric surgery shown in absolute values as the difference of bladder neck position at rest vs Valsalva maneuver 12.2 ± 6.7 vs 12.4 ± 6.6 (n/s, *p* = 0.34) (Fig. 5).

## Discussion

Obesity is considered as one of the most important risk factors of pelvic floor disorders. It is suggested that increased intra-abdominal pressure causes weakening of pelvic floor muscles and destruction of the fascia leading to pelvic organ prolapse and incontinence [13]. Moreover, obesity is associated with impairment of the quality of life (QOL) as far as pelvic floor symptoms are concerned [14].

Among pelvic floor disorders’ risk factors, only several are modifiable. There has been ongoing discussion to what extent delivery mode or elective cesarean section may prevent PFDs in high-risk subjects [15]. There are also strong evidences that pelvic floor muscle training improves POP and incontinence [16]. As far as the influence of weight loss on PFDs is concerned, it has been shown that bariatric surgery improves QOL and self-reported prolapse symptoms [17]. However, till now, there have been no reports concerning the influence of bariatric surgery on the anatomical and functional features of pelvic floor in patients without PFD. The questions seem to be of a great importance as possible protective effect of surgery-induced weight loss might become one of the important issues in patients’ counseling.

In the current study, for the first time, it was shown that BMI reduction results in the betterment of bladder neck position in patients without clinically manifested POP—one of the objective and measurable features of the pelvic floor anatomy. At the same time, we showed higher position of the bladder neck during tension of levator ani as well as during Valsalva maneuver. Most of current studies draw the conclusion about PFD improvement on the basis of patients’ questionnaires showing positive subjective results of bariatric surgery on the pelvic floor anatomy and function and they seem to be in agreement with our observations, which may be an objective explanation of such subjective improvement [18].

On the basis of the above observations, we postulate that weight loss may be a prophylaxis tool in the prevention of pelvic floor disorders probably in the mechanism of lowering of the intra-abdominal pressure. The above results might be an important argument when counseling patients before bariatric surgery.

On the other hand, we did not show any improvement in levator ani function measured as absolute value of the difference between bladder neck position at rest vs at levator ani tension. It may suggest that weight loss itself does not restore muscle function and other medical options should be considered.

The present work has also shown that bladder neck mobility measured as the difference of the bladder neck position at rest vs Valsalva maneuver was not restricted after bariatric surgery. It suggests that bladder neck hypermobility and descent that are often connected with urinary incontinence and POP do not change, and therefore, all abnormalities connected with pelvic floor injuries or weakening do not restore together with BMI normalization. It stays in agreement with observations showing no improvement of already existing POP symptoms after bariatric surgery [19].

In the current literature, there are strong evidences that weight loss results in at least partial resolution of incontinence symptoms. Nevertheless, it has been suggested that urinary incontinence in obese women is dependent rather on higher intra-vesical pressure than urethral hypermobility [20]. The above observation may explain incontinence improvement after bariatric surgery despite lack of restoration of bladder neck mobility that has been demonstrated in the current study.

All the above observations suggest that weight normalization after bariatric surgery improves anatomical features of the pelvic floor but at the same time does not change preexisting functional conditions. Therefore, it should be emphasized in the process of patients’ counseling that weight loss should result in betterment of pelvic floor anatomy as long as there is no serious impairment, but it will not restore muscle functions and preexisting fascia injuries resulted from, i.e., labors and obesity. Therefore, it should be taken into consideration that the sooner surgery-induced weight loss is obtained, the greater the chance for preserving pelvic floor wellness.

## Conclusions

Bariatric surgery is associated with a betterment of bladder neck position at rest, at tension, and during Valsalva maneuver in women without PFDs. On the basis of the above observations, we postulate that bariatric surgery may be a tool for PFD prevention resulting in bladder neck elevation probably through the reduction of intra-abdominal pressure. At the same time, it does not improve levator ani function and does not limit bladder neck mobility having no influence on preexisting pelvic floor functional features.
